# Clinical features and dysfunctions of iron metabolism in Parkinson disease patients with hyper echogenicity in substantia nigra: a cross-sectional study

**DOI:** 10.1186/s12883-018-1016-5

**Published:** 2018-01-17

**Authors:** Shu-yang Yu, Chen-jie Cao, Li-jun Zuo, Ze-jie Chen, Teng-hong Lian, Fang Wang, Yang Hu, Ying-shan Piao, Li-xia Li, Peng Guo, Li Liu, Qiu-jin Yu, Rui-dan Wang, Piu Chan, Sheng-di Chen, Xiao-min Wang, Wei Zhang

**Affiliations:** 1 Department of Geriatrics, Beijing Tiantan Hospital, Capital Medical University, Beijing, 100050 China; 2 Department of Neurology, Beijing Tiantan Hospital, Capital Medical University, Beijing, 100050 China; 3 Department of Neurobiology, Beijing Xuanwu Hospital, Capital Medical University, Beijing, 100053 China; 4 Center of Parkinson Disease, Beijing Institute for Brain Disorders, Beijing, 100069 China; 5 Beijing Key Laboratory on Parkinson Disease, Beijing, 100053 China; 6 Department of Neurology, Ruijin Hospital Affiliated to Shanghai Jiaotong University School of Medicine, Shanghai, 200025 China; 7 Department of Physiology, Capital Medical University, Beijing, 100069 China; 8 Key Laboratory for Neurodegenerative Disorders of the Ministry of Education, Capital Medical University, Beijing, 100069 China; 9 China National Clinical Research Center for Neurological Diseases, Beijing, 100050 China

**Keywords:** Parkinson disease, Transcranial sonography, Hyper echogenicity in substantia nigra, Dysfunction of iron metabolism

## Abstract

**Background:**

Transcranial ultrasound is a useful tool for providing the evidences for the early diagnosis and differential diagnosis of Parkinson disease (PD). However, the relationship between hyper echogenicity in substantia nigra (SN) and clinical symptoms of PD patients remains unknown, and the role of dysfunction of iron metabolism on the pathogenesis of SN hyper echogenicity is unclear.

**Methods:**

PD patients was detected by transcranial sonography and divided into with no hyper echogenicity (PDSN-) group and with hyper echogenicity (PDSN+) group. Motor symptoms (MS) and non-motor symptoms (NMS) were evaluated, and the levels of iron and related proteins in serum and cerebrospinal fluid (CSF) were detected for PD patients. Data comparison between the two groups and correlation analyses were performed.

**Results:**

PDSN+ group was significantly older, and had significantly older age of onset, more advanced Hohen-Yahr stage, higher SCOPA-AUT score and lower MoCA score than PDSN- group (*P* < 0.05). Compared with PDSN- group, the levels of transferrin and light-ferritin in serum and iron level in CSF were significantly elevated (P < 0.05), but ferroportin level in CSF was significantly decreased in PDSN+ group (*P* < 0.05).

**Conclusions:**

PD patients with hyper echogenicity in SN are older, at more advanced disease stage, have severer motor symptoms, and non-motor symptoms of cognitive impairment and autonomic dysfunction. Hyper echogenicity of SN in PD patients is related to dysfunction of iron metabolism, involving increased iron transport from peripheral system to central nervous system, reduction of intracellular iron release and excessive iron deposition in brain.

## Background

Parkinson disease (PD) is a neurodegenerative disease caused by a variety of factors,including aging, genetic predisposition and environment factors. PD patients have multiple non-motor symptoms (NMS), such as depression, constipation, rapid eye movement sleep behavior disorder and olfactory dysfunction, which can occur before the onset of motor symptoms (MS). Pathologically, PD was divided into six stages according to the regions that Lewy bodies deposit [1].When MS of PD patients occur, the pathology of the disease is at stage 3 and 4, which is featured by more than 50% of dopaminergic neuronal loss in SN and 70% of dopamine depletion in striatum, losing optimal chance of early treatment. It was found that in the premotor period, treatment before the significant neural degeneration might protect neurons against rapid deterioration, thereby delaying the disease progression [2]. Therefore, early diagnosis and treatment is a key strategy for PD patients.

Currently, positron emission tomography has an important value for the early diagnosis of PD, however, its limitations of high price, time-consuming, strict technical requirements and exposure to the tracer highly restrict its wide application clinically. Transcranial ultrasound (TCS), a non-invasive neuroimaging technique, is a useful tool for providing the evidences for the early diagnosis and differential diagnosis of PD. Although investigations on PD patients with TCS is increasing, however, most of them mainly focused on the relationship between hyper echogenicity in SN and disease duration or severity of MS [2–6]. In addition, there is still lack of studies about the relationship between NMS, particularly those that can occur before MS, and hyper echogenicity in SN. Hence, in-depth study of the relationship between NMS and echogenicity in SN of PD patients may provide clues to the early diagnosis of PD. In this study, a variety of rating scales were used to assess MS and NMS, and a comprehensive investigation on the association between clinical symptoms and echogenicity in SN were conducted in a relatively large PD population.

The mechanism of hyper echogenicity in SN of PD patient is unclear yet. Autopsies indicated that hyper echogenicity in SN might be relevant to excessive iron deposition in SN. However, the role of dysfunction of iron metabolism on hyper echogenicity in SN is rarely explored and elucidated. In this study, the levels of iron and its metabolism-related proteins in both serum and cerebrospinal fluid (CSF) from PD patients were measured, and their relationship with hyper echogenicity in SN were analyzed.

## Methods

This study was approved by Beijing Tiantan Hospital review board. Written informed consents were obtained from all participants in this study. All methods were performed in accordance with the relevant guidelines and regulations.

### Subjects

PD Patients were diagnosed according to UK Parkinson’s Disease Society Brain Bank criteria [7].Total 374 PD patients were consecutively recruited from the Department of Geriatrics and Neurology, Beijing Tiantan Hospital, Capital Medical University. Demographics variables, including gender, age, age of onset and disease duration were recorded. Among them, 122 cases finished all rating scales for clinical symptoms of PD, and 119 cases had CSF and blood samples for the detection of iron and related proteins. In 122 PD patients, 79 cases (64.8%) were male and 43 cases (35.2%) were female; patients’ age were 34~ 84 years with an average of 60.0 ± 10.5 years; disease duration was from 6 months to 33 years with an average of 2.0 (1.0~ 5.0) years.

### Detection of echogenicity and hyperechoic area of SN by TCS

Philips iU22 ultrasonic diagnostic apparatus equipped with S5-1 MHz phased array probe was used to detect echogenicity in SN with penetration depth of 14~ 16 cm and dynamic range of 45~ 55 dB. One experienced ultrasound practitioner who was responsible for detecting echogenicity in SN. The results were evaluated by the criteria proposed by Bartova P et al. [8] and divided into five grades. Grade I: the same as brainstem; grade II: with scattered points and thin lines slightly stronger than brainstem; grade III: with patches of moderate echogenicity but weaker than brain pool; grade IV: with patches of hyper echogenicity as the same as brain pool; grade V: with patches of hyper echogenicity stronger than brain pool.Grade I-II are defined as normal/SN-, and grade III-V as hyper echogenicity/SN+. PD patients with SN- were in PDSN- group and SN+ were in PDSN+ group, respectively. If the echo grades of two sides of SN were different, the higher one was chosen for the analyses. In some patients, there was only one temporal window available for detection of echogenicity in SN. Total 12 patients (9.0%) with both sides of temporal window unavailable were excluded and 122 cases were recruited in the investigation.

### Evaluations of clinical symptoms

Disease severity of PD is mainly reflected by Hohen-Yahr (H-Y) stage. MS was evaluated by Unified Parkinson’s Disease Rating Scale (UPDRS) III. NMS was assessed by a variety of rating scales, including Montreal Cognitive Assessment (MoCA) for cognitive impairment, Scale for Outcomes in PD For Autonomic Symptoms (SCOPA-AUT) for autonomic dysfunction, Hamilton Depression Scale (HAMD) -24 items for depression, Hamilton Anxiety Scale (HAMA)-14 items for anxiety, Pittsburgh Sleep Quality Index (PSQI) for sleep quality, Epworth Sleepiness Scale (ESS) for daytime sleepiness, Fatigue Scale (FS)-14 items for fatigue, Fatigue Severity Scale (FSS) for fatigue severity, and Restless Legs Syndrome (RLS) Severity Rating Scale (RLSRS) for RLS.

### Sample collections

Patients were requested to withhold anti-parkinsonian drugs for 12–14 h if their condition allowed. Total 2 ml venous whole blood was collected and 3 ml CSF was taken in a polypropylene tube between 7 a.m. and 10 a.m. under fasting condition through lumbar puncture, followed by being centrifuged in 4 °C at 3000 r/min for 10 min. Approximately 0.5 ml volume of serum and CSF were aliquoted into separate Nunc cryotubes and kept frozen at − 80 °C until ready for assay. Each aliquot dedicated for each measure to avoid freeze-thawing and potential degradation of protein.

### Detection of iron and related proteins

Iron and related proteins in serum and CSF from PD patients were detected by enzyme linked immunosorbent assay. We used Ab83366 kit from Abcam Company (British) for iron, CSB-E05187h kit for ferritin, CSB-E16932h for heavy-ferritin, CSB-E16933h for light-ferritin, Ab108911 kit for transferrin, CSB-E08387h for transferrin receptor, CSB-E08383 for ceruloplasmin and CSB-EL021641HU for ferroportin from Wuhan Huamei Biological Engineering Limited Company (China).

### Data analyses

Statistical analyses were performed with SPSS Statistics 21.0 (IBM Corporation, New York, USA). Demographic variables, disease features, the scores of MS and NMS, the levels of iron and related proteins in serum and CSF were all compared between PDSN- and PDSN+ groups. Continuous variables were presented as mean ± standard deviations and compared by 2-tailed T test if they were normally distributed, and presented as median (quartile) and compared by nonparametric test if they were not normally distributed. Discrete variables were compared by Chi square test. Spearman correlation analyses were made between echogenicityin SN and related factors above. *P* value was statistically significant when it was less than 0.05.

## Results

### The frequency of hyper echogenicity in SN of PD patients

**Fig. 1:**
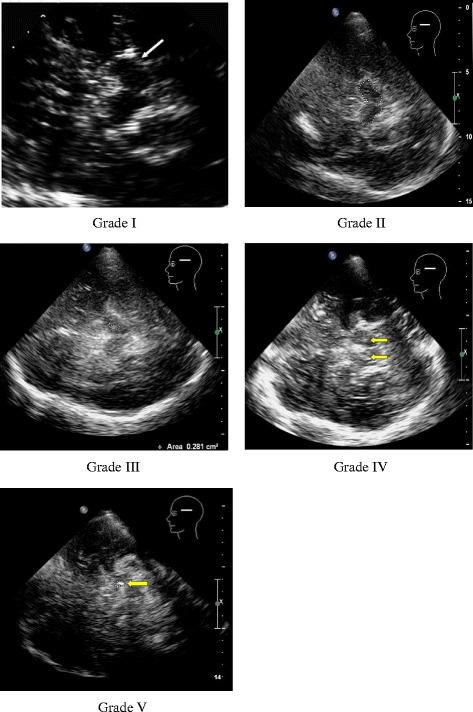
Grades of SN echogenicity by Transcranial ultrasound. Grade I: the same as brainstem; Grade II: with scattered points and thin lines slightly stronger than brainstem; Grade III: with patches of moderate echogenicity but weaker than brain pool; Grade IV: with patches of hyper echogenicity as the same as brain pool; Grade V: with patches of hyper echogenicity stronger than brain pool.

In this study, the frequency of hyper echogenicity in SN of PD patients was 68.2%. The cases of each grade of hyper echogenicity in SN were as followed: 0 case in grade I, 31 cases (25.4%) in grade II, 57 cases (46.7%) in grade III, 32 cases (26.2%) in grade IV and 2 cases in (1.6%) grade V. The picture of each grade of echogenicity was presented in SN (Fig. 1).

### Comparisons of demographic variables between PDSN+ and PDSN- groups

Male and older PD patients with later disease onset had higher incidence of hyper echogenicity in SN than female and younger ones with earlier disease onset (*P* < 0.05) (Table 1). Further correlation analysis showed that echogenicity in SN was significantly and positively correlated with age (*r* = 0.134, *P* = 0.011) and age of onset (*r* = 0.130, *P* = 0.014).

**Table 1:** Demographic variables and clinical symptoms of PDSN+ and PDSN- groups

Demographic features	PDSN- group	PDSN+ group	*P* value
Gender			0.028*
Male [case (%)]	15(48.4%)	64(70.3%)	
Female [case (%)]	16(51.6%)	27(29.7%)	
Age (year, mean ± SD)	54.3 ± 11.1	62.0 ± 9.6	0.002**
Age of onset (year, mean ± SD)	51.1 ± 10.9	57.9 ± 11.0	0.006**
Disease duration [year, median (quartile)]	2.0(1.0~ 4.0)	2.0(1.0~ 5.0)	0.919
MS			
H-Y stage [stage, median (quartile)]	2.0(1.0~ 5.0)	4.0(2.0~ 8.0)	0.004**
UPDRS III total score [score, median (quartile)]	21.6 ± 11.8	24.1 ± 13.6	0.467
MNS			
Cognitive impairment			
MoCA (score, mean ± SD)	22.7 ± 5.8	20.1 ± 5.4	0.047*
Visuospatial and execution[score, median(quartile)]	3.0(2.0~ 5.0)	3.0(2.0~ 4.0)	0.992
Naming [score, median (quartile)]	3.0(3.0~ 3.0)	3.0(2.0~ 3.0)	0.045*
Memory [score, median (quartile)]	3.0(0~ 4.0)	3.0(0~ 3.0)	0.038*
Attention and calculation [score, median (quartile)]	6.0(4.5~ 6.0)	6.0(4.0~ 6.0)	0.067
Language [score, median (quartile)]	2.0(2.0~ 3.0)	2.0(2.0~ 3.0)	0.953
Abstraction [score, median (quartile)]	1.0(1.0~ 2.0)	1.0(1.0~ 2.0)	0.408
Orientation [score, median (quartile)]	6.0(6.0~ 6.0)	6.0(5.0~ 6.0)	0.709
Autonomic symptoms			
Digestive function [score, median (quartile)]	8.0(7.0~ 10.0)	10.0(9.0~ 11.0)	0.001**
Urination function [score, median (quartile)]	7.0(6.0~ 9.0)	10.0(8.0~ 13.0)	0.000**
Cardiovascular function [score, median(quartile)]	3.5(3.0~ 4.0)	4.0(3.0~ 6.0)	0.067
Temperature regulation (score, mean ± SD)	5.7 ± 1.8	6.5 ± 2.2	0.116
Pupil adjustment [score, median(quartile)]	1.0(1.0~ 2.0)	1.0(1.0~ 2.0)	0.047*
Sexual function (score, mean ± SD)	3.0 ± 1.5	4.0 ± 2.0	0.175
SCOPA-AUT total score (score, mean ± SD)	33.5 ± 7.7	36.8 ± 9.0	0.044*
Emotional disorders			
HAMD [score, median (quartile)]	10.0(3.0~ 16.0)	10.0(5.0~ 17.0)	0.538
HAMA [score, median (quartile)]	8.0(4.0~ 18.0)	11.0(5.0~ 17.0)	0.623
Sleep disorder			
PSQI [score, median (quartile)]	6.0(2.0~ 12.0)	5.0(2.0~ 7.0)	0.446
ESS [score, median (quartile)]	6.0(3.0~ 9.0)	4.0(2.0~ 8.0)	0.829
Fatigue			
FS-14 (score, mean ± SD)	8.6 ± 4.4	8.2 ± 3.8	0.565
FSS (score, mean ± SD)	36.2 ± 15.7	39.6 ± 16.5	0.307
Restless leg syndrome			0.494
No [case(%)]	20(64.5%)	64(70.3%)	
Yes [case(%)]	11(35.5%)	27(29.7%)	

*P < 0.05,**P < 0.01

### Comparisons of clinical symptoms between PDSN+ and PDSN- groups

PDSN+ group had significantly more advanced H-Y stage than PDSN- group (P < 0.05). The scores of naming and memory in PDSN+ group were significantly decreased compared with PDSN- group (Table 1). Autonomic dysfunction, including digestive, urinary and pupil adjusting symptoms were also prominently compromised in PDSN+ group than PDSN- group (Table 1). However, the scores of depression, anxiety, sleep disorder, fatigue and restless leg syndrome were not significantly different between two groups (Table 1). Correlations of echogenicity in SN with H-Y stage and UPDRS III score were not significant (*P* > 0.05), but SN echogenicity was significantly and negatively correlated with MoCA score (*r* = − 0.189,*P* = 0.001), negatively correlated with urinary symptoms (*r* = 0.157, *P* = 0.004) and pupil adjusting symptoms (*r* = 0.119, *P* = 0.028) in SCOPA-AUT. Further multiple linear regression analysis between SN echogenicity and related factors showed that memory and urinary symptoms were independently associated with echogenicity in SN (Table 2).

**Table 2:** Multiple linear regression analysis between SN hyper echogenicity and related factors

Related Factors	□*β*	*P*	∆R^2^	Adjusted R^2^
Memory	−0.51	0.010	0.031	0.027
Urinary symptoms	0.26	0.030	0.049	0.041

### Comparisons of iron and related proteins in CSF and serum between PDSN+ and PDSN- groups

In CSF, iron level from PDSN+ group was significantly higher than that from PDSN- group (Table 3). However, ferroportin level in CSF was significantly lower than that from PDSN- group.

**Table 3:** The levels of iron and related proteins in CSF in PDSN+ and PDSN- groups

Iron and Related Proteins	PDSN- group (29 cases)	PDSN+ group (90 cases)	*P* value
Iron (ng/ml)	0.57(0.34~ 0.92)	0.89(0.42~ 3.32)	0.015*
Tf (ng/ml)	0.12(0.10~ 0.26)	0.13(0.11~ 0.19)	0.612
TfR1 (ng/ml)	228.07(106.35~ 264.98)	161.11(123.83~ 215.75)	0.134
Cp (ng/ml)	0.37(0.24~ 0.58)	0.24(0.11~ 0.51)	0.082
Fpn (pg/ml)	43.70(33.86~ 63.30)	35.31(23.39~ 51.65)	0.034*
Fer (ng/ml)	5.77(0.86~ 15.63)	2.08(1.12~ 11.11)	0.231
H-Fer (ng/ml)	1.10(0.80~ 1.60)	1.14(0.88~ 1.61)	0.743
L-Fer (ng/ml)	1.19(0.94~ 2.35)	1.29(0.87~ 1.56)	0.609

*P < 0.05

In serum, **t**he levels of transferrin and light-ferritin from PDSN+ group were both significantly higher than that from PDSN- group (Table 4).

**Table 4:** The levels of iron and related proteins in serum in PDSN+ and PDSN- groups

Iron and Related Proteins	PDSN- group (29 cases)	PDSN+ group (90 cases)	*P* value
Iron(nmol/l)	2.86(2.19~ 4.33)	3.04(2.10~ 4.40)	0.689
Tf(nmol/l)	0.12(0.09~ 0.17)	0.15(0.11~ 0.19)	0.009*
TfR1(ng/ml)	266.78(169.60~ 360.50)	292.75(213.17~ 346.77)	0.294
Cp(ng/ml)	0.45(0.30~ 2.68)	0.46(0.32~ 3.02)	0.495
Fpn(pg/ml)	76.56(59.26~ 119.04)	85.24(57.48~ 126.75)	0.563
Fer(ng/ml)	21.30(12.03~ 54.68)	19.65(9.75~ 61.70)	0.470
H-Fer(ng/ml)	1.99(1.34~ 2.74)	2.23(1.47~ 2.91)	0.132
L-Fer(ng/ml)	2.10(1.41~ 2.80)	2.45(1.77~ 3.10)	0.047*

*P < 0.05

### Correlation analyses of the levels of iron and related proteins between CSF and serum

The levels of iron and related proteins, including ferritin, light chain ferritin and transferrin in CSF were significantly and positively correlated with that in serum (*r* = 0.182 and *P* = 0.030 for iron, *r* = 0.538 and *P* = 0.001 for ferritin, *r* = 0.292 and *P* = 0.001 for light chain ferritin, and *r* = 0.444 and *P* = 0.001 for transferrin).

## Discussions

Hyperechoic phenomenon in SN of PD patients was firstly observed by Becker et al. [9]. However, the relationship between hyper echogenicity in SN and demographic information in PD patient is not clear yet. In this study, comparisons of the demographic variables between PDSN+ group and PDSN- group suggested that male, old age and late disease onset were relevant to hyper echogenicity in SN of PD patients. Although the reason for the gender difference was rarely explored, estrogen was thought to be involved [10, 11]. Estrogen was found to make female individuals less prone to iron accumulation by reducing iron levels [12], and hyper echogenicity in SN was associated with elevated unbound iron [13–15], thus female individuals more likely have lower echogenicity in SN. Here, it was also found that older PD patients with late disease onset have higher echogenicity in SN, which might be due to the increasingly accumulation of brain iron during aging process.

In this study, echogenicity in SN was not related to disease duration, which was supported by another study observing no significant change in hyperechoic area even with an interval of 8 years between examinations [16]. These results illucidate that disease duration is not a definitive factor for the iron accumulation and related hyper echogenicity in SN of PD patients.

In this investigation, echogenicity in SN was associated disease severity indicated by the advanced H-Y stage. In our previous study, it was found that iron induced dopaminergic neurodegeneration through neuroinflammatoty mechanism indicated by the over activation of microglia and robust production of neurotoxic factors [17], which might explain hyper echogenicity revealed by the elevated iron level in SN was correlated with the rapid progression of PD reflected by advanced H-Y stage.

Comparison of non-motor symptoms of PD between PDSN+ group and PDSN- group. Firstly, the data showed that MoCA score in the former group was significantly lower than that in the later group. The cognitive domains of naming and memory evaluated by MoCA scale were seriously impaired in PDSN+ group compared with PDSN- group. Further correlation analyses and multiple linear regression analysis revealed that memory decline was independently associated with echogenicity in SN of PD patients. There are two dopaminergic pathways in the brain, midbrain-limbic pathway and mesencephalic-limbic system-cortical pathway. The midbrain-limbic pathway starts from dopamine neurons in the medial ventral tegmental area and the medial part of substantia nigra pars compact (SNpc). The mesencephalic-limbic system-cortical pathway also contains the dopamine neurons in SNpc. These two neural pathways are thought to be related to cognitive function, which damage decreases the level of dopamine and thus causes impairments of multiple cognitive domains, such as memory, attention and execution [18]. Hence, depletion of dopamine relevant to hyper echogenicity in SN was correlated to cognitive dysfunction in PD population.

Secondly, autonomic dysfunction, including gastrointestinal, urinary and oculopupillary symptoms, in PDSN+ group were significantly prominent compared with PDSN- group. Further correlation analyses and multiple linear regression analysis revealed urinary symptom was independently associated with echogenicity in SN of PD patients. Symptoms of a dysautonomia can be found in the early stage of PD patients. Pathological studies demonstrated that α-synuclein, the main component of Lewy bodies, first appeared in the low brainstem, such as vagus dorsal nucleus in medullary, was responsible for the clinical symptoms of early autonomic dysfunction [1]. Manifestations of dysautonomia together with hyper echogenicity in SN may offer pivotal cues for early prediction and diagnosis of PD.

In normal condition, iron is necessary for the maintenance of physiological function of neurons. However, excessive iron deposition in brain may cause a cascade events of oxidative stress and neuroinflammation that destroy neuronal phospholipid membranes, proteins and nucleic acids, leading to the degeneration and death of neurons [19]. Thus, appropriate iron level in brain is vital for maintaining a stable internal environment through a rigorous regulatory mechanism.

In this study, iron level in CSF from PDSN+ group was significantly higher than that from PDSN- group. According to the autopsy and animal investigations, iron level in CSF reflected its level in brain. The levels of transferrin and light-ferritin in serum from PDSN+ group were both significantly higher than that from PDSN- group. The levels of iron and related proteins, including ferritin, light chain ferritin and transferrin in CSF were significantly and positively correlated with that in serum. Iron level in CSF was positively correlated with serum iron, ferritin, light chain ferritin and transferrin. Elevated serum iron, ferritin and light chain ferritin could reflect an increase of total iron in serum due to excessive iron intake; and elevated transferrin in serum may suggest that more serum iron is transported from blood to the brain. Ferroportin is responsible for iron transport from inside to outside of cells [20], and its reduction in CSF may lead to a less intracellular iron excretion and consequently excessive iron deposition in brain.

## Conclusions

PD patients with hyper echogenicity in SN have older age, more advanced disease stage, more severe motor non-motor symptoms. Hyper echogenicity in SN in PD patients is related to dysfunction of iron metabolism, involving increased iron intake, transport from peripheral to central nervous system and reduction of intracellular iron release. Non-motor symptoms, such as mild cognitive impairment and autonomic dysfunctions, can occur in early stage of PD, which is related with hyper echogenicity in SN, thus demonstrating that combinations of cognitive impairment, autonomic dysfunction and hyper echogenicity in SN may serve as potential biomarker for early prediction and diagnosis of PD.

There is a limitation of this study, which is the lack of age-matched healthy control due to the great difficulty of obtaining CSF samples from such population. We will try our best to collect the samples from healthy control in the future in order to compare dysfunction of iron metabolism and echogenicity in SN between PD and normal subjects.

## Abbreviations

AHRS: Argentina Hyposmia Rating Scale
CSF: cerebrospinal fluid
HAMD: Hamilton Depression Scale
H-Y: Hohen-Yahr
MS: Motor symptoms
NMS: non-motor symptoms
PD: Parkinson disease
RBD: Rapid Eye Movement Behavior Disorder
RBDSQ: Rapid Eye Movement Behavior Disorder Screening Questionnaire
RLS: Restless Legs Syndrome
RLSRS: Restless Legs Syndrome Severity Rating Scale
SCOPA-AUT: Scale for Outcomes in PD For Autonomic Symptoms
SN: substantia nigra
TCS: Transcranial ultrasound
UPDRS: Unified Parkinson’s Disease Rating Scale
